# Fosmanogepix (APX001) Is Effective in the Treatment of Pulmonary Murine Mucormycosis Due to Rhizopus arrhizus

**DOI:** 10.1128/AAC.00178-20

**Published:** 2020-05-21

**Authors:** Teclegiorgis Gebremariam, Sondus Alkhazraji, Abdullah Alqarihi, Nathan P. Wiederhold, Karen Joy Shaw, Thomas F. Patterson, Scott G. Filler, Ashraf S. Ibrahim

**Affiliations:** a The Lundquist Institute for Biomedical Innovations at Harbor-University of California Los Angeles (UCLA) Medical Center, Torrance, California, USA; b University of Texas Health Science Center at San Antonio, San Antonio, Texas, USA; c Hearts Consulting Group, San Diego, California, USA; d South Texas Veterans Health Care System, San Antonio, Texas, USA; e David Geffen School of Medicine at UCLA, Los Angeles, California, USA

**Keywords:** APX001, APX001A, Gwt1, antifungal, *Rhizopus arrhizus*, infection model, mucormycosis, 1-aminobenzotriazole, manogepix, MGX, fosmanogepix, antifungal agents, infectious disease

## Abstract

Mucormycosis is a life-threatening infection with high mortality that occurs predominantly in immunocompromised patients. Manogepix (MGX) is a novel antifungal that targets Gwt1, a protein involved in an early step in the conserved glycosylphosphotidyl inositol (GPI) posttranslational modification pathway of surface proteins in eukaryotic cells. Inhibition of fungal inositol acylation by MGX results in pleiotropic effects, including inhibition of maturation of GPI-anchored proteins necessary for growth and virulence. MGX has been previously shown to have *in vitro* activity against some strains of Mucorales. Here, we assessed the *in vivo* activity of the prodrug fosmanogepix, currently in clinical development for the treatment of invasive fungal infections, against two Rhizopus arrhizus strains with high (4.0 μg/ml) and low (0.25 μg/ml) minimum effective concentration (MEC) values. In both invasive pulmonary infection models, treatment of mice with 78 mg/kg or 104 mg/kg fosmanogepix, along with 1-aminobenzotriazole to enhance the serum half-life of MGX in mice, significantly increased median survival time and prolonged overall survival by day 21 postinfection compared to placebo. In addition, administration of fosmanogepix resulted in a 1 to 2 log reduction in both lung and brain fungal burden. For the 104 mg/kg fosmanogepix dose, tissue clearance and survival were comparable to clinically relevant doses of isavuconazole (ISA), which is FDA approved for the treatment of mucormycosis. These results support continued development of fosmanogepix as a first-in-class treatment for invasive mucormycosis.

## Abstract (precis)

Mucormycosis is a life-threatening infection with high mortality that occurs predominantly in immunocompromised patients. Manogepix (MGX) is a novel antifungal that targets Gwt1, a protein involved in an early step in the conserved glycosylphosphotidyl inositol (GPI) posttranslational modification pathway of surface proteins in eukaryotic cells. Inhibition of fungal inositol acylation by MGX results in pleiotropic effects, including inhibition of maturation of GPI-anchored proteins necessary for growth and virulence.

## INTRODUCTION

Mucormycosis is an often-lethal infection that occurs predominantly in immunocompromised patients, including those suffering from diabetic ketoacidosis (DKA) or neutropenia. Due to the rising prevalence of diabetes, cancer, and organ transplantation in the aging population worldwide, the number of patients at risk for this deadly infection is on the rise (1–3). In addition, analysis of invasive mold infections from blast injuries in military personnel in Afghanistan showed that among 31 patients, Mucorales and *Aspergillus* spp. were the predominant organisms isolated (16 patients each), followed by *Fusarium* spp. (9 patients) (4). Unfortunately, despite disfiguring surgical debridement and adjunctive antifungal therapy, the overall mortality of mucormycosis remains >50% (5), and this rate approaches 100% in patients with disseminated disease (6). Thus, new strategies to prevent and treat mucormycosis are urgently needed.

Glycosylphosphatidylinositol (GPI)-anchored proteins are known to have several functions, including mediating adhesion to and invasion of host tissues by microorganisms (7, 8). Therefore, they play a pivotal role in pathogenesis of infectious diseases. Fosmanogepix (formerly APX001 and E1210) is the prodrug of manogepix (MGX, formerly APX001A and E1210), a broad-spectrum investigational antifungal agent that inhibits inositol acyltransferase, thereby preventing GPI-anchored protein maturation (9). This inhibition results in pleiotropic effects on fungal growth and virulence.

Previous studies have shown that MGX has broad *in vitro* activity against *Candida* spp., *Aspergillus* spp., and some rare molds (9–11). The MGX MEC values against limited numbers of Mucorales strains were more moderate and variable, with reported ranges of 0.12 to 16 μg/ml against Rhizopus arrhizus; 2 to 16 μg/ml against R. microsporus; 1 to 16 μg/ml against Rhizomucor pusillus; and 0.5 to 8 μg/ml against Mucor circinelloides using CLSI methodology (9, 12). These MEC values are similar to the ranges of MIC values seen for isavuconazole (ISA) in a study of worldwide isolates from 2015 to 2016 conducted by two reference laboratories (13). In that study, a wide range in MIC values was observed as follows: 0.25 to 4 μg/ml against R. arrhizus; 0.5 to 32 μg/ml against R. microsporus; 0.5 to 8 μg/ml against Rhizomucor pusillus; and 2 to 32 μg/ml against M. circinelloides (13).

The correlation between MIC or MEC values and outcome in clinical studies has not been well established, especially for values >16 μg/ml (14). However, correlations in MIC (*Candida*) or MEC (*Aspergillus*) values have generally been observed in pharmacokinetic/pharmacodynamic (PK/PD) mouse studies that evaluated isolates with MGX MIC or MEC values below 0.125 μg/ml (15). Thus, we sought to assess the activity of fosmanogepix in neutropenic murine invasive pulmonary mucormycosis (16) models using two strains of *Rhizopus*, the most common cause of mucormycosis, in which the MEC values of MGX were low (0.25 μg/ml for R. arrhizus var. *delemar*) and high (4.0 μg/ml for R. arrhizus var. arrhizus).

We utilized fosmanogepix treatment regimens in mice that resulted in MGX exposures (the area under the plasma drug concentration-time curve [AUC]) similar to what was observed in phase 1 clinical studies (15, 17, 18). This was accomplished by preadministration of 50 mg/kg 1-aminobenzotriazole (ABT), a nonselective suicide inhibitor of cytochrome P450 (CYP) enzymes (19). We have previously shown that ABT administered 2 h prior to fosmanogepix enhanced MGX exposures 16- to 18-fold and enhanced serum half-life from ∼1 to 9 h, more closely mimicking human pharmacokinetic values (2 to 2.5 days) observed in phase 1 clinical studies in healthy volunteers (15, 17, 18). As a comparator in the efficacy studies, we orally administered the prodrug isavuconazonium sulfate (ISA) using a dosing regimen that resulted in isavuconazole exposures in mice similar to what has been observed in human clinical studies (20, 21). ISA was utilized as the comparator since it was approved by the FDA in 2015 for the treatment of invasive aspergillosis and invasive mucormycosis (CRESEMBA [isavuconazonium sulfate]). In addition, ISA was approved by the European Medicines Agency (EMA) for treating patients with mucormycosis who cannot be treated with amphotericin B. The efficacy endpoints in the mouse models were survival and reduction in fungal burden in target organs.

## RESULTS

### Antifungal susceptibility.

The microbiological activities of MGX, posaconazole (POSA, another antifungal drug used to treat mucormycosis), and ISA were evaluated against 17 and 19 clinical isolates of R. arrhizus var. arrhizus and R. arrhizus var. *delemar*, respectively. MGX MEC interpretive criteria for molds were as described for the echinocandins and MIC values were determined for the comparators ISA and POSA (22). R. arrhizus var. *delemar* strains were more susceptible to MGX than R. arrhizus var. arrhizus, with MEC geometric means (GM) of 0.75 μg/ml and 3.84 μg/ml, respectively (Table 1). In contrast, R. arrhizus var. arrhizus isolates were more sensitive to ISA (GM = 0.85 μg/ml) than R. arrhizus var. *delemar* strains (GM = 2.5 μg/ml). Both sets of clinical isolates demonstrated similar susceptibility to POSA with GMs of 0.15 and 0.36 μg/ml for R. arrhizus var. arrhizus and R. arrhizus var. *delemar*, respectively (Table 1). The MGX MEC values for the two clinical isolates used in the animal models were 0.25 μg/ml and 4 μg/ml for R. arrhizus var. *delemar* strain 99-880 and R. arrhizus var. arrhizus strain 99-892, respectively, representing a 16-fold difference between the two strains. For both isolates, the MIC values for POSA was 0.125 μg/ml, while the ISA MICs were 2.0 μg/ml for R. arrhizus var. arrhizus 99-892 and 1.0 μg/ml for R. arrhizus var. *delemar* 99-880. The selection of these two strains allowed examination of the efficacy of fosmanogepix against *Rhizopus* infections where the strains demonstrated high and low MEC values, compared to a control drug (ISA) where the two strains demonstrated similar MIC values (1 and 2 μg/ml, for isolates 99-880 and 99-892, respectively).

**TABLE 1:** Antifungal susceptibility of clinical isolates of R. arrhizus var. *delemar* and R. arrhizus var. arrhizus^*a*^

Isolate	Assessment type	MEC or MIC in μg/ml		
		MGX	POSA	ISA
*R. arrhizus* var. *delemar* (*n* = 19)	Range	0.25–8.0	0.125–1.0	1.0–8.0
	MEC/MIC_50_	0.5	0.25	2.0
	MEC/MIC_90_	4.0	1.0	4.0
	GM MEC/MIC	0.75	0.36	2.5
*R. arrhizus* var. *arrhizus* (*n* = 17)	Range	0.25–8.0	0.06–0.5	0.25–2.0
	MEC/MIC_50_	8.0	0.125	1.0
	MEC/MIC_90_	8.0	0.25	1.0
	GM MEC/MIC	3.84	0.15	0.85

a Readings were taken as the minimum effective concentration (MEC) for MGX and MIC_50_ or MIC_90_ for POSA and ISA. MGX, manogepix; ISA, isavuconazole; POSA, posaconazole; GM, geometric mean.

### Fosmanogepix demonstrates efficacy in a highly immunosuppressed mouse model of pulmonary mucormycosis caused by R. arrhizus var. *delemar*.

In order to assess fosmanogepix in our established model of neutropenic murine mucormycosis, ICR mice were immunosuppressed with cyclophosphamide (200 mg/kg) and cortisone acetate (500 mg/kg) on days −2, +3, and +8, relative to intratracheal infection with 2.5 × 10^5^ cells of R. arrhizus var. *delemar* 99-880 on day 0 (17). Treatment with placebo (diluent control), fosmanogepix (52, 78, or 104 mg/kg, once daily orally [p.o.]), or ISA (110 mg/kg three times a day [TID], p.o.) began 16 h postinfection and continued for 7 days. To extend the half-life of MGX in mice, 50 mg/kg of the cytochrome P450 inhibitor 1-aminobenzotriazole (ABT) was administered 2 h prior to fosmanogepix administration, as previously described (23). Control mice did not receive ABT because ABT has no *in vitro* activity against *Rhizopus* strains and we previously demonstrated no difference in survival of mice infected with pulmonary invasive aspergillosis and treated with or without ABT (24). ISA was dosed at 110 mg/kg p.o. TID to achieve an exposure in mice that is equivalent to clinical exposures achieved in humans (20, 21). Similarly, ABT plus fosmanogepix (78 mg/kg and 104 mg/kg once daily) achieved exposures in mice that approximate exposures achieved in clinical doses in phase 1 trials (17, 18), whereas the 52 mg/kg dose achieved a lower exposure.

**FIG 1:**
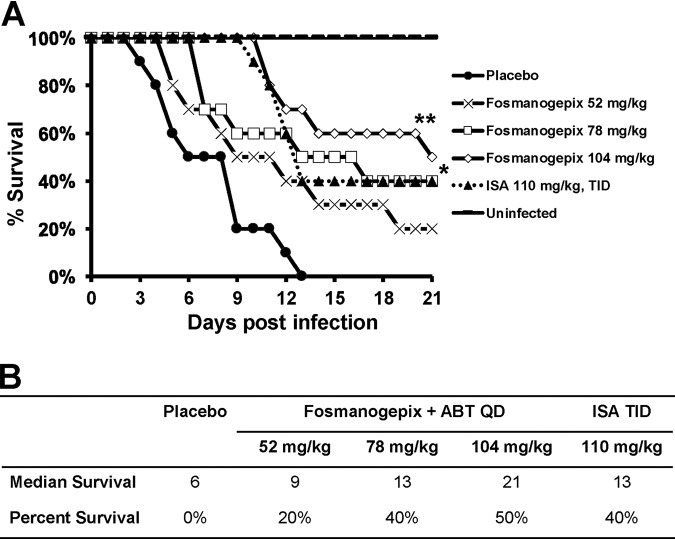
Survival of immunosuppressed mice infected with R. arrhizus var. delemar 99-880 (low MEC). Mice (*n* = 10/group) were infected intratracheally with R. arrhizus var. *delemar* (inhaled inoculum of 7 × 10^3^ spores/mouse) and 16 h later treated with ISA 110 mg/kg TID p.o., or with fosmanogepix QD p.o. for 7 days. ABT was administered 2 h prior to each fosmanogepix treatment to enhance the half-life of MGX in mice. *, *P = *0.001 for 78 mg/kg fosmanogepix and 110 ISA; **, *P* < 0.0001 for 104 mg/kg fosmanogepix versus placebo mice by log rank test. (A) Kaplan-Meier survival curve. (B) Median and percent survival at day 21.

**(i) Survival**. Mice survival was assessed over 21 days (*n* = 10 mice/cohort). Doses of fosmanogepix in mice that achieved clinically relevant exposures (78 mg/kg and 104 mg/kg) demonstrated significantly improved survival compared to the placebo control (*P < *0.01 by log rank test), whereas the low dose of 52 mg/kg fosmanogepix, although numerically better than placebo control, did not achieve significance (*P = *0.07) (Fig. 1A). As we previously reported (20), a clinically relevant exposure of ISA in mice also improved survival compared to placebo (*P = *0.001), but was not significantly different from any of the three fosmanogepix dosing groups (*P > *0.25) (Fig. 1A). The two highest dosing groups of fosmanogepix resulted in 40% and 50% survival at day 21 postinoculation, respectively, similar to ISA (40% survival at this time point) (Fig. 1A). Furthermore, fosmanogepix demonstrated a dose-dependent prolongation in median survival time of mice (9, 13, and 21 days) versus placebo (6 days). The median survival time for ISA was 13 days (Fig. 1B).

**FIG 2:**
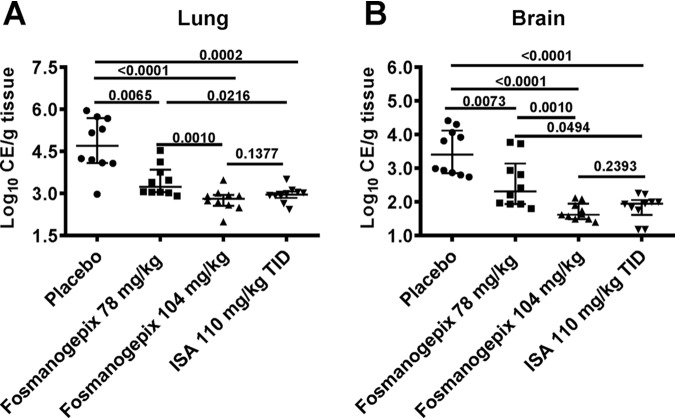
Reduction in tissue fungal burden of immunosuppressed mice infected with R. arrhizus var. *delemar* 99-880. Mice (*n* = 10/group) infected intratracheally with R. arrhizus var. *delemar* (inhaled inoculum of 1.1 × 10^4^ spores/mouse) and 16 h later treated with ISA 110 mg/kg TID p.o., or with fosmanogepix 104 mg/kg QD p.o. On day +4, organs were collected and processed for tissue fungal burden by qPCR. Data are presented as the median ± interquartile range and the *y* axis represents the lower limit of detection. Intergroup *P* values are shown as a dark line. All dosing groups resulted in a statistically significant reduction in brain and lung fungal burden versus placebo control using the Wilcoxon rank sum test.

**(ii) Tissue fungal burden**. Mice were immunosuppressed and infected as described above; however, mice (*n* = 10/group) were sacrificed at day +4 after infection to determine conidial equivalents (CE)/g of lung and brain tissue by quantitative PCR (qPCR) using 18S primers. In the placebo group, fungal burdens (CE) at day +4 were log_10_ 4.74 ± 0.96 (lung) and log_10_ 3.48 ± 0.67 (brain) (Fig. 2). For the 78 mg/kg and 104 mg/kg fosmanogepix dosing groups, a 1.3 and 1.97 log_10_ reduction in CE/g of lung tissue was observed, respectively, which was similar to what was observed for ISA (1.79 log_10_ reduction in CE). Reductions in log_10_ CE/g of brain tissue were 0.93, 1.78, and 1.65 for 78 mg/kg fosmanogepix, 104 mg/kg fosmanogepix, and 110 mg/kg TID ISA, respectively. In all three treatment groups, the CE counts observed for tissue fungal burden in lung and brains were significantly lower than the placebo control group (Fig. 2). However, the 104 mg/kg fosmanogepix dosing regimen reduced tissue fungal burden to a greater degree than the 78 mg/kg fosmanogepix treatment (*P = *0.001), and was equivalent to tissue burden reductions observed for the ISA treatment (*P > *0.13).

### Fosmanogepix demonstrates efficacy in a highly immunosuppressed mouse model of pulmonary mucormycosis caused by R. arrhizus var. arrhizus.

The efficacy of fosmanogepix was assessed in the immunosuppressed murine pulmonary mucormycosis model using a strain of R. arrhizus var. arrhizus that had a 16-folder higher MEC value than the R. arrhizus var. *delemar* 99-880 strain. ICR mice were immunosuppressed and infected intratracheally as above with 2.5 × 10^5^ spores of R. arrhizus var. arrhizus 99-892/mouse on day 0 (with confirmed lung-delivered inoculum of 1.1 × 10^4^ spores). Due to the better performance of 104 mg/kg fosmanogepix versus the 78 mg/kg treatment group in the R. arrhizus var. *delemar* mucormycosis model, only the higher dosing regimen was evaluated versus ISA in this efficacy model.

**FIG 3:**
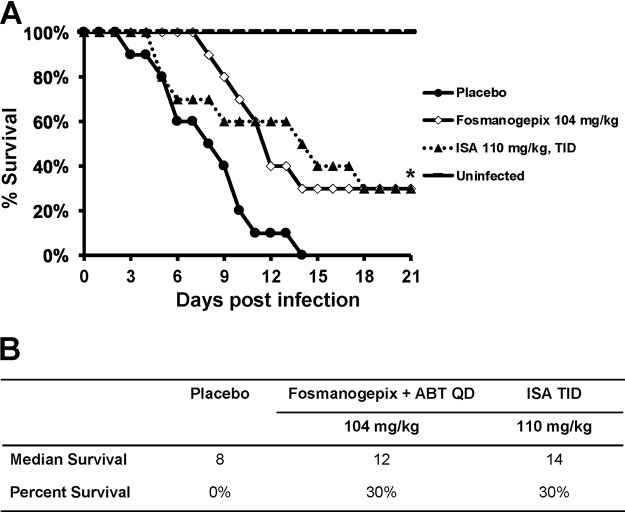
Survival of immunosuppressed mice infected with R. arrhizus var. arrhizus 99-892 (high MEC). Mice (*n* = 10/group) infected intratracheally with R. arrhizus var. arrhizus (inhaled inoculum of 1.1 × 10^4^ spores/mouse) and 16 h later treated with ISA 110 mg/kg TID p.o., or with fosmanogepix 104 mg/kg QD p.o. for 7 days. ABT was administered 2 h prior to fosmanogepix treatment to enhance the half-life of manogepix in mice. *, *P* = 0.01 for 104 mg/kg fosmanogepix; *P* = 0.02 for ISA 110 mg/kg, TID versus placebo mice by log rank test. (A) Kaplan-Meier survival curve. (B) Median and percent survival at day 21.

**(i) Survival.** In the survival model, mice (*n* = 10 mice/cohort), were assessed for 21 days. Both treatments of 104 mg/kg fosmanogepix or ISA (110 mg/kg TID, PO) demonstrated efficacy in this mucormycosis model with 30% overall survival compared to 0% survival for placebo-treated mice (*P* < 0.05 versus placebo control [Fig. 3]). Despite the difference in MEC/MIC values against this strain, fosmanogepix (4 μg/ml) and ISA (2 μg/ml) survival curves were not significantly different from each other (*P* = 0.80) (Fig. 3).

**FIG 4:**
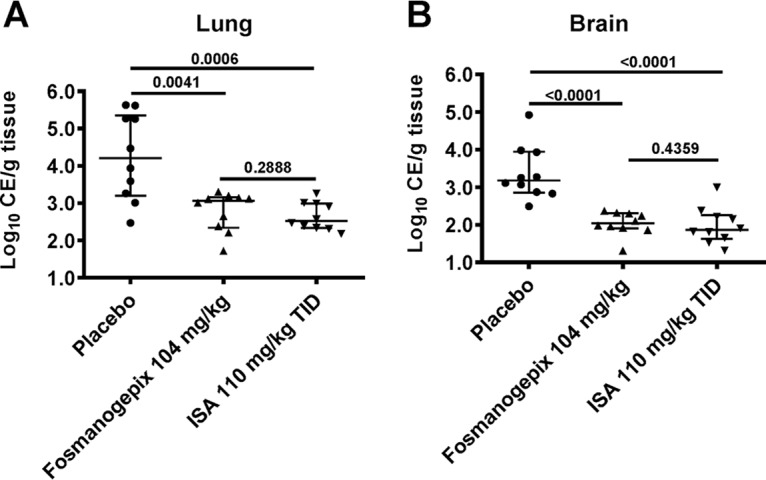
Reduction in tissue fungal burden of immunosuppressed mice infected with R. arrhizus var. arrhizus 99-892. Mice (*n* = 10/group) infected intratracheally with R. arrhizus var. arrhizus (inhaled inoculum of 6.4 × 10^3^ spores/mouse) and 16 h later treated with ISA 110 mg/kg TID p.o., or with fosmanogepix 104 mg/kg QD p.o. On day +4, organs were collected and processed for tissue fungal burden by qPCR. Data are presented as the median ± interquartile range and the *y* axis represents the lower limit of detection. Intergroup *P* values are shown as a dark line. Both fosmanogepix and ISA resulted in a statistically significant reduction in brain and lung fungal burden versus placebo control using the Wilcoxon rank sum test.

**(ii) Tissue fungal burden.** Mice were infected and treated as above but sacrificed on day +4 postinfection. Lung and brain tissue were harvested to determine conidial equivalents (CE)/g of lung and brain tissue by qPCR using 18S primers. Fungal burdens at day +4 for the placebo group were log_10_ 4.21 ± 1.0 (lung) and 3.18 ± 0.4 log_10_ (brain) (Fig. 4). Compared to placebo, both fosmanogepix and ISA treatment groups demonstrated significant reductions in lung (*P ≤ *0.001) and brain (*P* < 0.0001) CE. Treatment with 104 mg/kg fosmanogepix reduced lung and brain CE by log_10_ 1.15 (lung) and log_10_ 1.14 (brain), whereas ISA reduced CE by log_10_ 1.69 (lung) and log_10_ 1.14 (brain). ISA and fosmanogepix CE reductions were not significantly different (*P* > 0.28) for either brain or lung.

## DISCUSSION

Although relatively rare, mucormycosis is difficult to treat and is frequently life-threatening, with all-cause mortality estimated to be 54% (25). These infections are most often seen in solid organ and stem cell transplant recipients, diabetics, neutropenic patients, or patients treated with corticosteroids. Guidelines for the treatment of mucormycoses recommend high dose liposomal amphotericin B, posaconazole, or isavuconazole as first line therapy, and these infections often require surgical debridement and immune recovery to improve the chances of a successful outcome (26). Isavuconazole (administered as the prodrug isavuconazonium sulfate, CRESEMBA) is approved for the treatment of adults with invasive mucormycosis. In a phase 3, open-label, noncomparative trial (VITAL) for isavuconazole, the most common organisms identified were Rhizopus arrhizus (*oryzae*) and Mucormycetes (27). Of 22 Mucorales isolated and evaluated during the VITAL trial, ISA MIC values ranged from 0.25 μg/ml for a single strain of Actinomucor elegans to 32 μg/ml for some strains of *M. circinelloides* and *Rhizopus* species (27). These values are similar to the range of ISA MIC values (0.25 to ≥16 μg/ml) observed for 292 Mucorales obtained from 2015 and 2016 at two reference laboratories (13). Although no correlations were observed between trough ISA plasma concentrations, MIC values, and key outcomes, the authors indicate that this is possibly due to the relatively small number of patients in the study. Similarly, Andes et al. evaluated the efficacy of ISA and voriconazole against *Aspergillus* spp. and found that although the drugs were clearly ineffective against strains where MICs were ≥16 μg/ml, there was no clear relationship with clinical outcomes in cases where the MIC was <16 μg/ml for either drug (14).

Fosmanogepix is an intravenously (i.v.) and orally (p.o.) available antifungal prodrug that is currently in clinical development for the treatment of life-threatening invasive fungal infections. The broad-spectrum activity of this first-in-class agent has been demonstrated *in vivo* using multiple mouse models of invasive pulmonary and disseminated fungal infections, including those of *Candida* spp., *Coccidioides* spp., *Cryptococcus* spp., *Aspergillus* spp., *Fusarium* spp., and *Scedosporium* spp. (15, 24, 28–31). These studies demonstrated increased survival, reduced fungal burden in lung, kidney, and brain tissues of infected mice, as well as histological improvement (15, 23, 30, 32, 33). Here, we extend these findings to *Rhizopus*, where we evaluated the efficacy against two different strains with MGX MEC values of 0.25 μg/ml and 4.0 μg/ml.

We previously evaluated the efficacy of ISA in murine models of pulmonary mucormycosis due to R. arrhizus var. *delemar* 99-880 or *M. circinelloides* and demonstrated improved mouse survival and clearance of fungal burden versus placebo control (20, 34). In this study, as a comparator in both experiments, we also utilized 110 mg/kg ISA (TID), a dose which gives rise to exposures in mice that are similar to exposures achieved clinically by ISA. MIC values of ISA against R. arrhizus var. *delemar* 99-880 and R. arrhizus var. arrhizus 99-892 were similar (1.0 and 2.0 μg/ml, respectively). Similar to our historical data (20, 34), in this study ISA significantly improved survival and reduced fungal burden in brain and lung by 1 to 2 log reductions versus the placebo control. Importantly, in the current study, similar results were seen for the 104 mg/kg fosmanogepix cohorts in both infection models. Total exposures for the 78 mg/kg and 104 mg/kg doses in mice (14) are ∼200 and ∼280 μg · h/ml, respectively (K. J. Shaw, unpublished data) and were previously shown to be associated with A. fumigatus stasis and 1 log kill in PK/PD studies (28). These exposures in mice are consistent with exposures achieved in fosmanogepix phase 1 single and multiple ascending-dose studies (17, 18) and anticipated in future fosmanogepix clinical trials. Thus, although the MGX MEC values of the two strains differed by 16-fold (0.25 versus 4.0 μg/ml), the efficacy of fosmanogepix at clinically relevant total exposures was equivalent to ISA, which is approved for the treatment of mucormycosis. Fosmanogepix has now been shown to be efficacious against both varieties of Rhizopus arrhizus, which together are the main cause of the disease and responsible for ∼50% of cases of lethal mucormycosis (25).

In this study, we used a highly immunocompromised mouse model to show that fosmanogepix has equivalent efficacy to ISA, a current standard of care antifungal drug, in treating pulmonary mucormycosis. Findings from both oral and i.v. fosmanogepix phase 1 clinical studies have shown favorable PK, allowing once-daily dosing, with high bioavailability (∼90%), and no food effect (17, 18). These data support further investigations into the development of this first-in-class agent for a broad range of difficult-to-treat invasive fungal infections.

## MATERIALS AND METHODS

### Antifungal agents.

For *in vitro* studies, the active moiety manogepix (Amplyx Pharmaceuticals), ISA active compound, and POSA (both from Sigma-Aldrich Corp. St. Louis, MO, USA) were used. For efficacy studies, the water-soluble *N*-phosphonooxymethyl prodrug fosmanogepix (Amplyx Pharmaceuticals) was dissolved in a final prodrug solution of 5% dextrose. The water soluble isavuconazonium sulfate (Astellas Pharam US) was purchased from Bellavida Pharmacy, Torrance, CA. POSA (Merck & Co., Inc., Rahway, NJ) was purchased as an oral suspension (200 mg/5 ml) and kept at room temperature. All drugs were prepared fresh and orally (p.o.) dosed per gram mouse body weight on a daily basis. A 5 mg/ml solution of ABT (Fisher Scientific, Hampton, NH) in water was administered orally 2 h prior to administration of fosmanogepix at 10 μl per gram mouse body weight resulting in a dose of 50 mg/kg.

### Microorganisms.

R. arrhizus var. *delemar* 99-880 and R. arrhizus var. arrhizus 99-892 are brain and lung isolates obtained from the Fungus Testing Laboratory at the University of Texas Health Sciences Center at San Antonio (UTHSCSA). Other strains from R. arrhizus var. *delemar* and R. arrhizus var. arrhizus were also obtained from the culture collection of the Fungus Testing Laboratory, and species identification was confirmed as previously described (13). Strains were routinely grown on potato dextrose agar plates for 4 days until confluent at 37°C. Spores were collected by flooding the plates with sterile phosphate-buffered saline (PBS) containing 0.01% (vol/vol) Tween 80. The spores were concentrated by centrifugation, washed in the same buffer, diluted, and counted using a hemocytometer.

### *In vitro* testing.

The *in vitro* susceptibility to manogepix, ISA, and POSA by fungal agents of mucormycosis was evaluated using the Clinical Laboratory and Standards Institute (CLSI) M38 method using minimum effective concentration (MEC) endpoints, as per the echinocandins, and MIC endpoints for POSA and ISA (22).

### Efficacy models.

The pulmonary mucormycosis model has been previously described (16). Briefly, ICR mice (Envigo) were immunosuppressed with cyclophosphamide (200 mg/kg) and cortisone acetate (500 mg/kg) on days −2, +3, and +8 relative to infection. This regimen was shown to result in ∼16 days of leucopenia (16). To prevent bacterial infection, 50 μg/ml enrofloxacin (Baytril; Bayer, Leverkusen, Germany) was added to the drinking water from day −3 to day 0. Ceftazidine (5 μg/dose/0.2 ml) replaced enrofloxacin treatment on day 0 and was administered daily by subcutaneous injection from day 0 until day +4 (tissue fungal burden) or day +13 (survival). Mice were challenged with R. arrhizus var. *delemar* or R. arrhizus var. arrhizus (2.5 × 10^5^/mouse) through intratracheal instillation of 25 μl after sedation with isoflurane gas (16). Immediately after infection, a subset of mice was sacrificed and lungs were removed to determine conidial equivalent (CE) by qPCR. All drug treatments (by oral gavage) were initiated at 16 h postinfection and continued for 7 consecutive days (survival) or 4 days (tissue burden assessment). To extend the half-life of MGX after fosmanogepix administration, 50 mg/kg of ABT was administered 2 h prior to each daily fosmanogepix dose. The prodrug fosmanogepix was dosed at 78 mg/kg and 104 mg/kg once daily by oral gavage. Using a conversion factor of 1.3 to account for the methyl phosphate group in the prodrug, the doses were equivalent to MGX at 60 mg/kg and 80 mg/kg, respectively. ISA was dosed at 110 mg/kg TID, the dose that gives rise to exposures equivalent to the human clinical dose (21). For the survival experiments, mice were monitored for 21 days. To assess tissue fungal burden, mice were sacrificed on day +4 and organs processed for conidial equivalent (CE) by real-time qPCR using 18S primers (sense amplification primer, 5′-GCGGATCGCATGGCC-3′; antisense amplification primer, 5′-CCATGATAGGGCAGAAAATCG-3′.)

### Ethics statement.

All animal-related study procedures were compliant with the Animal Welfare Act, the Guide for the Care and Use of Laboratory Animals, and the Office of Laboratory Animal Welfare and were conducted under an IACUC-approved protocol by The Lundquist Institute for Biomedical Innovations at Harbor-UCLA Medical Center.

### Statistical analyses.

The nonparametric log rank test was used to determine differences in survival times. Differences in lung and brain CE were compared by the nonparametric Wilcoxon rank sum test. All analyses were corrected for multiple comparisons with the Bonferroni correction. A *P* value of <0.05 was considered significant.

## References

[1] Marr KA, Carter RA, Crippa F, Wald A, Corey L 2002 Epidemiology and outcome of mould infections in hematopoietic stem cell transplant recipients. Clin Infect Dis 34:909–917. doi:10.1086/339202. 11880955

[2] Kontoyiannis DP, Wessel VC, Bodey GP, Rolston KV 2000 Zygomycosis in the 1990s in a tertiary-care cancer center. Clin Infect Dis 30:851–856. doi:10.1086/313803. 10852735

[3] Kauffman CA 2004 Zygomycosis: reemergence of an old pathogen. Clin Infect Dis 39:588–590. doi:10.1086/422729. 15356828

[4] Warkentien T, Rodriguez C, Lloyd B, Wells J, Weintrob A, Dunne JR, Ganesan A, Li P, Bradley W, Gaskins LJ, Seillier-Moiseiwitsch F, Murray CK, Millar EV, Keenan B, Paolino K, Fleming M, Hospenthal DR, Wortmann GW, Landrum ML, Kortepeter MG, Tribble DR, Infectious Disease Clinical Research Program Trauma Infectious Disease Outcomes Study G 2012 Invasive mold infections following combat-related injuries. Clin Infect Dis 55:1441–1449. doi:10.1093/cid/cis749. 23042971 PMC3657499

[5] Sugar AM 1995 Agent of mucormycosis and related species, p 2311–2321. *In* Mandell G, Bennett J, Dolin R (ed), Principles and practices of infectious diseases, 4th ed Churchill Livingstone, New, York, NY.

[6] Husain S, Alexander BD, Munoz P, Avery RK, Houston S, Pruett T, Jacobs R, Dominguez EA, Tollemar JG, Baumgarten K, Yu CM, Wagener MM, Linden P, Kusne S, Singh N 2003 Opportunistic mycelial fungal infections in organ transplant recipients: emerging importance of non-*Aspergillus* mycelial fungi. Clin Infect Dis 37:221–229. doi:10.1086/375822. 12856215

[7] Fu Y, Ibrahim AS, Sheppard DC, Chen YC, French SW, Cutler JE, Filler SG, Edwards JE. Jr., 2002 *Candida albicans* Als1p: an adhesin that is a downstream effector of the EFG1 filamentation pathway. Mol Microbiol 44:61–72. doi:10.1046/j.1365-2958.2002.02873.x. 11967069

[8] Gebremariam T, Liu M, Luo G, Bruno V, Phan QT, Waring AJ, Edwards JE Jr., Filler SG, Yeaman MR, Ibrahim AS 2014 CotH3 mediates fungal invasion of host cells during mucormycosis. J Clin Invest 124:237–250. doi:10.1172/JCI71349. 24355926 PMC3871245

[9] Miyazaki M, Horii T, Hata K, Watanabe NA, Nakamoto K, Tanaka K, Shirotori S, Murai N, Inoue S, Matsukura M, Abe S, Yoshimatsu K, Asada M 2011 *In vitro* activity of E1210, a novel antifungal, against clinically important yeasts and molds. Antimicrob Agents Chemother 55:4652–4658. doi:10.1128/AAC.00291-11. 21825291 PMC3186989

[10] Castanheira M, Duncanson FP, Diekema DJ, Guarro J, Jones RN, Pfaller MA 2012 Activities of E1210 and comparator agents tested by CLSI and EUCAST broth microdilution methods against *Fusarium* and *Scedosporium* species identified using molecular methods. Antimicrob Agents Chemother 56:352–357. doi:10.1128/AAC.05414-11. 22083469 PMC3256086

[11] Pfaller MA, Hata K, Jones RN, Messer SA, Moet GJ, Castanheira M 2011 *In vitro* activity of a novel broad-spectrum antifungal, E1210, tested against *Candida* spp. as determined by CLSI broth microdilution method. Diagn Microbiol Infect Dis 71:167–170. doi:10.1016/j.diagmicrobio.2011.05.001. 21696907

[12] Rivero-Menendez O, Cuenca-Estrella M, Alastruey-Izquierdo A 2019 *In vitro* activity of APX001A against rare moulds using EUCAST and CLSI methodologies. J Antimicrob Chemother 74:1295–1299. doi:10.1093/jac/dkz022. 30753499

[13] Pfaller MA, Rhomberg PR, Wiederhold NP, Gibas C, Sanders C, Fan H, Mele J, Kovanda LL, Castanheira M 2018 *In vitro* activity of isavuconazole versus opportunistic fungal pathogens from two mycology reference laboratories. Antimicrob Agents Chemother 62:e01230-18. doi:10.1128/AAC.01230-18. 30061288 PMC6153788

[14] Andes DR, Ghannoum MA, Mukherjee PK, Kovanda LL, Lu Q, Jones ME, Santerre Henriksen A, Lademacher C, Hope WW 2018 Outcomes by MIC values for patients treated with isavuconazole or voriconazole for invasive aspergillosis in the phase 3 SECURE and VITAL trials. Antimicrob Agents Chemother 63:e01634-18. doi:10.1128/AAC.01634-18. 30373791 PMC6325202

[15] Zhao M, Lepak AJ, VanScoy B, Bader JC, Marchillo K, Vanhecker J, Ambrose PG, Andes DR 2018 In vivo pharmacokinetics and pharmacodynamics of APX001 against *Candida* spp. in a neutropenic disseminated candidiasis mouse model. Antimicrob Agents Chemother 62:e02542-17. doi:10.1128/AAC.02542-17. 29378706 PMC5913987

[16] Luo G, Gebremariam T, Lee H, French SW, Wiederhold NP, Patterson TF, Filler SG, Ibrahim AS 2013 Efficacy of liposomal amphotericin B and posaconazole in intratracheal models of murine mucormycosis. Antimicrob Agents Chemother 57:3340–3347. doi:10.1128/AAC.00313-13. 23650163 PMC3697351

[17] Hodges MR, Ople E, Shaw KJ, Mansbach RS, van Marle S, van Hoogdalem E, Kramer W, Wedel P 2017 Phase 1 study to assess safety, tolerability and pharmacokinetics of single and multiple oral doses of APX001 and to investigate the effect of food on APX001 bioavailability, abstr 1860. IDweek 2017, San Diego, CA https://idsa.confex.com/idsa/2017/webprogram/Paper64127.html.

[18] Hodges MR, Ople E, Shaw KJ, Mansbach RS, van Marle S, van Hoogdalem E, Wedel P, Kramer W 2017 First-in-human study to assess safety, tolerability and pharmacokinetics of APX001 administered by intravenous infusion to healthy subjects, abstr 1840. IDweek 2017, San Diego, CA https://idsa.confex.com/idsa/2017/webprogram/Paper63677.html.

[19] Balani SK, Zhu T, Yang TJ, Liu Z, He B, Lee FW 2002 Effective dosing regimen of 1-aminobenzotriazole for inhibition of antipyrine clearance in rats, dogs, and monkeys. Drug Metab Dispos 30:1059–1062. doi:10.1124/dmd.30.10.1059. 12228180

[20] Luo G, Gebremariam T, Lee H, Edwards JE Jr., Kovanda L, Ibrahim AS 2014 Isavuconazole therapy protects immunosuppressed mice from mucormycosis. Antimicrob Agents Chemother 58:2450–2453. doi:10.1128/AAC.02301-13. 24492363 PMC4023778

[21] Lepak AJ, Marchillo K, Vanhecker J, Diekema D, Andes DR 2013 Isavuconazole pharmacodynamic target determination for Candida species in an *in vivo* murine disseminated candidiasis model. Antimicrob Agents Chemother 57:5642–5648. doi:10.1128/AAC.01354-13. 24002092 PMC3811283

[22] Clinical and Laboratory Standards Institute. 2017 Reference method for broth dilution antifungal susceptibility testing of filamentous fungi. M38, approved standard, third edition, Clinical and Laboratory Standards Institute, Wayne, PA.

[23] Zhao Y, Lee MH, Paderu P, Lee A, Jimenez-Ortigosa C, Park S, Mansbach RS, Shaw KJ, Perlin DS 2018 Significantly improved pharmacokinetics enhances in vivo efficacy of APX001 against echinocandin- and multidrug-resistant *Candida* isolates in a mouse model of invasive candidiasis. Antimicrob Agents Chemother 62:e00425-18. doi:10.1128/AAC.00425-18. 30012766 PMC6153843

[24] Gebremariam T, Alkhazraji S, Alqarihi A, Jeon HH, Gu Y, Kapoor M, Shaw KJ, Ibrahim AS 2018 APX001 is effective in the treatment of murine invasive pulmonary aspergillosis. Antimicrob Agents Chemother 63:e01713-18. doi:10.1128/AAC.01713-18. PMC6355556 30455236

[25] Roden MM, Zaoutis TE, Buchanan WL, Knudsen TA, Sarkisova TA, Schaufele RL, Sein M, Sein T, Chiou CC, Chu JH, Kontoyiannis DP, Walsh TJ 2005 Epidemiology and outcome of zygomycosis: a review of 929 reported cases. Clin Infect Dis 41:634–653. doi:10.1086/432579. 16080086

[26] Cornely OA, Alastruey-Izquierdo A, Arenz D, Chen SCA, Dannaoui E, Hochhegger B, Hoenigl M, Jensen HE, Lagrou K, Lewis RE, Mellinghoff SC, Mer M, Pana ZD, Seidel D, Sheppard DC, Wahba R, Akova M, Alanio A, Al-Hatmi AMS, Arikan-Akdagli S, Badali H, Ben-Ami R, Bonifaz A, Bretagne S, Castagnola E, Chayakulkeeree M, Colombo AL, Corzo-León DE, Drgona L, Groll AH, Guinea J, Heussel C-P, Ibrahim AS, Kanj SS, Klimko N, Lackner M, Lamoth F, Lanternier F, Lass-Floerl C, Lee D-G, Lehrnbecher T, Lmimouni BE, Mares M, Maschmeyer G, Meis JF, Meletiadis J, Morrissey CO, Nucci M, Oladele R, Pagano L, Pasqualotto A, Patel A, Racil Z, Richardson M, Roilides E, Ruhnke M, Seyedmousavi S, Sidharthan N, Singh N, Sinko J, Skiada A, Slavin M, Soman R, Spellberg B, Steinbach W, Tan BH, Ullmann AJ, Vehreschild JJ, Vehreschild MJGT, Walsh TJ, White PL, Wiederhold NP, Zaoutis T, Chakrabarti A, Mucormycosis ECMM MSG Global Guideline Writing Group 2019 Global guideline for the diagnosis and management of mucormycosis: an initiative of the European Confederation of Medical Mycology in cooperation with the Mycoses Study Group Education and Research Consortium. Lancet Infect Dis 19:e405–e421. doi:10.1016/S1473-3099(19)30312-3. 31699664 PMC8559573

[27] Marty FM, Ostrosky-Zeichner L, Cornely OA, Mullane KM, Perfect JR, Thompson GR 3rd, Alangaden GJ, Brown JM, Fredricks DN, Heinz WJ, Herbrecht R, Klimko N, Klyasova G, Maertens JA, Melinkeri SR, Oren I, Pappas PG, Racil Z, Rahav G, Santos R, Schwartz S, Vehreschild JJ, Young JA, Chetchotisakd P, Jaruratanasirikul S, Kanj SS, Engelhardt M, Kaufhold A, Ito M, Lee M, Sasse C, Maher RM, Zeiher B, Vehreschild MJGT, VITAL and FungiScope Mucormycosis Investigators 2016 Isavuconazole treatment for mucormycosis: a single-arm open-label trial and case-control analysis. Lancet Infect Dis 16:828–837. doi:10.1016/S1473-3099(16)00071-2. 26969258

[28] Zhao M, Lepak AJ, Marchillo K, Vanhecker J, Sanchez H, Ambrose PG, Andes DR 2019 APX001 pharmacokinetic/pharmacodynamic target determination against *Aspergillus fumigatus* in an *in vivo* model of invasive pulmonary aspergillosis. Antimicrob Agents Chemother 63:e02372-18. doi:10.1128/AAC.02372-18. 30670426 PMC6437477

[29] Viriyakosol S, Kapoor M, Okamoto S, Covel J, Soltow QA, Trzoss M, Shaw KJ, Fierer J 2018 APX001 and other Gwt1 inhibitor prodrugs are effective in experimental *Coccidioides immitis* pneumonia. Antimicrob Agents Chemother 63:e01715-18. doi:10.1128/AAC.01715-18. PMC6355600 30455238

[30] Shaw KJ, Schell WA, Covel J, Duboc G, Giamberardino C, Kapoor M, Moloney M, Soltow QA, Tenor JL, Toffaletti DL, Trzoss M, Webb P, Perfect JR 2018 *In vitro* and *in vivo* evaluation of APX001A/APX001 and other Gwt1 inhibitors against *Cryptococcus*. Antimicrob Agents Chemother 62:e00523-18. doi:10.1128/AAC.00523-18. 29891599 PMC6105804

[31] Alkhazraji S, Gebremariam T, Alqarihi A, Gu Y, Mamouei Z, Singh S, Wiederhold NP, Shaw KJ, Ibrahim AS 2019 Fosmanogepix (APX001) is effective in the treatment of immunocompromised mice infected with invasive pulmonary scedosporiosis or disseminated fusariosis. Antimicrob Agents Chemother 64:e01735-19. doi:10.1128/AAC.01735-19. PMC7038288 31818813

[32] Hata K, Horii T, Miyazaki M, Watanabe NA, Okubo M, Sonoda J, Nakamoto K, Tanaka K, Shirotori S, Murai N, Inoue S, Matsukura M, Abe S, Yoshimatsu K, Asada M 2011 Efficacy of oral E1210, a new broad-spectrum antifungal with a novel mechanism of action, in murine models of candidiasis, aspergillosis, and fusariosis. Antimicrob Agents Chemother 55:4543–4551. doi:10.1128/AAC.00366-11. 21788462 PMC3187015

[33] Hager CL, Larkin EL, Long L, Zohra Abidi F, Shaw KJ, Ghannoum MA 2018 *In vitro* and *in vivo* evaluation of the antifungal activity of APX001A/APX001 against *Candida auris*. Antimicrob Agents Chemother 62:e02319-17. doi:10.1128/AAC.02319-17. 29311065 PMC5826120

[34] Gebremariam T, Wiederhold NP, Alqarihi A, Uppuluri P, Azie N, Edwards JE Jr., Ibrahim AS 2017 Monotherapy or combination therapy of isavuconazole and micafungin for treating murine mucormycosis. J Antimicrob Chemother 72:462–466. doi:10.1093/jac/dkw433. 27798213 PMC6289498

